# Software Choice Matters in Mutational Signature Refitting

**DOI:** 10.3390/biology15201899

**Published:** 2026-10-21

**Authors:** Jan Sliacky, Monika Kovacova

**Affiliations:** Faculty of Informatics and Informational Technologies, Slovak University of Technology, Ilkovicova 2, 541 01 Bratislava, Slovakia

**Keywords:** mutational signatures, signature refitting, TRACERx

## Abstract

Background: Mutational signature refitting is widely used to quantify the contributions of known mutagenic processes in cancer genomes. However, different software implementations can yield discordant results even when applied to the same data. Methods: This study compares five commonly used refitting tools—Sigminer, MutationalPatterns, sigfit, musicatk, and SigProfilerAssignment—across two independent datasets: TRACERx whole-exome sequencing data and a Genome in a Bottle (GIAB) reference sample. Inter-tool agreement was evaluated using six complementary metrics: Frobenius distance, Manhattan distance, cosine similarity, Pearson correlation, covariance-based distance, and spectral distance. Results: Across all metrics, musicatk and Sigminer demonstrated the strongest concordance, while sigfit was the most divergent. This ranking was consistent in the single-sample GIAB analysis, suggesting that the observed discrepancies arise primarily from implementation differences between tools rather than from dataset-specific factors. Conclusions: Our findings underscore the importance of cross-tool validation and careful consideration of tool-dependent variability when mutational signatures are used for biological interpretation or biomarker development.

## Simple Summary

Mutational signatures are DNA change patterns used widely in cancer research and diagnostics, yet it is often unclear how similar the results are across different software implementations. In this study, we compared five commonly used tools on two public datasets and found notable differences: musicatk and Sigminer produced the most consistent results, while sigfit differed the most. Because the same pattern across tools appeared in both datasets, the variation mainly reflects differences between the tools themselves. This highlights that software choice can strongly affect mutational signature analysis, and that cross-tool validation is important for experimental reproducibility.

## 1. Introduction

Somatic mutations accumulate in human cells throughout life as a consequence of multiple endogenous and exogenous mutational processes, including spontaneous DNA damage, replication errors, defective DNA repair, and exposure to environmental mutagens. Each process leaves a characteristic pattern of genomic alterations, commonly referred to as a mutational signature, which can be detected in cancer genomes and used to infer the biological mechanisms that generated these mutations [1,2,3]. Mutational signature analysis has therefore become a central approach in cancer genomics, because it enables the decomposition of tumor mutation catalogues into latent mutagenic processes and their relative contributions within individual samples.

In its standard form, mutational signature analysis represents each tumor as a mixture of signatures defined over mutation categories, most commonly in 96 trinucleotide combinations of single-base substitutions. Within this framework, a signature corresponds to a probability distribution over mutation types, whereas the exposure quantifies how strongly a given process contributes to a particular genome [3]. This paradigm has been widely used in both de novo extraction and refitting settings and has provided important insights into mutational processes associated with aging, defective homologous recombination, mismatch repair deficiency, APOBEC activity, ultraviolet radiation, tobacco smoke, and therapy-related mutagenesis [1,2,3,4]. Large-scale studies such as pan-cancer analysis of human genome (PCAWG) established mutational signatures as a standard component of cancer genome analysis and expanded the catalogue of reference signatures now widely used across research and translational applications [2].

Despite the broad adoption of mutational signature analysis, the computational inference of signatures is not methodologically uniform. Existing software packages rely on different mathematical and statistical frameworks, including classical non-negative matrix factorization (NMF), Bayesian NMF, hierarchical probabilistic models, and related inference strategies. Even within NMF-based approaches, the underlying optimization problem does not guarantee a unique optimal solution, and practical results depend on choices such as the number of extracted signatures, initialization, regularization, convergence criteria, and attribution strategy [3]. Some algorithms incorporate stochastic search procedures, meaning that repeated runs on the same data may yield different solutions unless analysis settings are tightly controlled [2].

These methodological differences are especially important because mutational signature decomposition is not a purely automatic or deterministic procedure. Prior work has emphasized that extracting signatures often requires model selection, sensitivity analysis, and biological plausibility checks, particularly for flat or highly correlated signatures that are mathematically difficult to separate [2,3]. The stability of decomposition can vary markedly across signatures, and certain signatures are much less robust to perturbation than others. As a consequence, analyses performed with different software tools, parameter settings, or random initializations may not converge on the same biological interpretation.

This variability has direct implications for reproducibility. If different mutational signature tools produce discordant outputs because of distinct underlying algorithms and non-deterministic inference behavior, then downstream conclusions about mutagenic exposures, DNA repair defects, or clinically relevant biomarkers may also differ. Such inconsistency is problematic for bioinformatics benchmarking, for cross-study comparison in cancer genomics, and for potential clinical use cases in which signature-based biomarkers are linked to treatment response, such as homologous recombination deficiency and PARP inhibitor sensitivity. Here, we focus on systematic comparison of mutational signature software which we believe is necessary to assess how algorithmic design influences output concordance, robustness, and reproducibility in research healthcare setting.

### Organization of This Study

The Introduction defined mutational signatures as characteristic patterns of somatic mutations associated with distinct mutagenic processes and distinguishes de novo signature extraction, in which both signatures and exposures are inferred from the data, from signature refitting, in which exposures are estimated using a predefined reference catalogue.

Section 2 then describes the experimental design, the TRACERx 100 whole-exome sequencing cohort and the Genome in a Bottle whole-genome reference sample, together with mutation preprocessing and catalogue construction. This section also introduces the five evaluated refitting tools, namely Sigminer, MutationalPatterns, sigfit, musicatk, and SigProfilerAssignment, and explains the harmonization and matrix-comparison procedures.

Section 3 presents pairwise cross-tool comparisons for the TRACERx and GIAB datasets using complementary distance- and similarity-based measures.

Section 4 discusses the observed agreement and disagreement between tools, the consistency of the results across datasets, and the implications of algorithm-dependent variability for reproducibility and biological interpretation.

Finally, Section 5 summarizes the main conclusions and emphasizes that mutational-signature results should be interpreted cautiously and, where possible, validated across multiple computational tools rather than relying on a single implementation.

## 2. Materials and Methods

### 2.1. Experimental Design

We designed a set of experiments to extract mutational signatures from cancer omics data and compared the results across several software tools. Dozens of de novo and refitting methods exist, many are outdated or no longer maintained. We therefore elected five actively used tools: four R packages—Sigminer (v2.3.0) [5], sigfit (v2.2.0) [6], MutationalPatterns (v3.22.0) [7] and musicatk (v2.6.0) [8]—and the Python tool SigProfilerAssignment (v1.1.3) [9]. Because all can perform both de novo extraction and refitting, we focused our experiments on refitting and restricted the analysis to single-base substitution signatures. The method choice was decided as Non-Negative Matrix Factorization (NMF). These tools use NMF when calculating the signatures de novo. For refitting, they use Non-negative Least Squares except for sigfit which uses Markov Chain Monte Carlo algorithm. The results for respective software tools were compared using six different comparison methods, namely Frobenius distance, Manhattan distance, Cosine similarity, Correlation-based metric, Covariance-based distance and Spectral distance.

### 2.2. Experimental Data

We assembled our own TRACERx 100 subset, consisting of whole-exome sequencing from the first 100 TRACERx patients (EGAS00001002247), totaling 427 tumor WES samples and ~14.4 TB of aligned binary Illumina short-read data. Because this dataset contains only exomes, we added Genome in a Bottle [10] as a complementary whole-genome dataset to assess whether WGS behaves differently. The downloaded BAM files were originally aligned to GRCh37, so we remapped them to GRCh38 using GATK 4.4.0.0 with BWA-MEM. The vcf files were generated using HaplotypeCaller and VQSR filtering was applied at a 90.0 threshold [11], and removed decoy chromosomes before downstream signature analysis. After filtering, the overall mutation distribution remained largely unchanged, although the total number of mutations dropped substantially (Figure 1). As a second dataset, we used Genome in a Bottle, which was left unfiltered; the GIAB sample contains 3,893,341 mutations. As a signature library, the respective in-built libraries were used (e.g., SBS_hg38 for sigminer) or COSMIC_v3.2 [12].

### 2.3. Mutational Signatures and Refitting Principle

Mutational signatures are characteristic patterns of somatic mutations that reflect the biological processes acting on a cancer genome. These processes may include spontaneous DNA damage, replication errors, defective DNA repair, tobacco exposure, ultraviolet radiation, and cellular aging. In the most commonly used single-base substitution framework, mutations are classified into 96 mutation types according to six substitution classes—C > A, C > G, C > T, T > A, T > C, and T > G—and the nucleotide context immediately preceding and following the mutated base. This SBS96 representation provides sufficient resolution to distinguish mutational processes with different biological origins while remaining computationally tractable.

Mathematically, a mutation catalogue can be represented as a non-negative combination of mutational signatures and their exposures:

M ≈ S × E

where (*M*) denotes the observed mutation catalogue, (*S*) is the matrix of mutational signatures, and (*E*) is the exposure matrix. Each column of (*S*) describes the mutation-type distribution associated with one mutational process, whereas each element of (*E*) quantifies the contribution of that process to a particular sample.

Two principal analytical strategies are used in mutational-signature analysis. In de novo extraction, both the signature matrix (*S*) and the exposure matrix (*E*) are estimated from the observed mutation catalogues. This approach can identify signatures that are not included in existing reference catalogues, but it requires a sufficiently large number of mutations and is sensitive to the selected model, initialization, number of components, and optimization procedure. In contrast, signature refitting assumes that (*S*) is known in advance from a curated reference catalogue, such as COSMIC, and estimates only the exposure matrix (*E*) what makes the problem computationally less problematic. Refitting is therefore more appropriate for sparse whole-exome sequencing data, for which the number of observed mutations is generally insufficient for reliable de novo discovery.

In the present study, we focused on SBS signature refitting rather than de novo extraction. The same TRACERx dataset and a comparable refitting strategy were previously used to investigate whether mutational signatures could identify tobacco exposure in whole-exome sequencing data [1]. Here, we extended this methodological perspective by focusing specifically on the concordance of the outputs produced by different refitting tools. The five tools examined in this study use related but not identical computational procedures: most estimate non-negative exposures using non-negative least squares or quadratic programming, whereas sigfit applies a Bayesian Markov chain Monte Carlo framework. These algorithmic differences provide the basis for assessing how software choice affects the resulting exposure matrices.

### 2.4. Mutation-Signature Extraction Tools and Algorithms

Since each tool has its own configuration, we applied targeted fine-tuning for each one, as outlined below.

**Sigminer**: To build the decomposition matrix, we used *sig_tally()* function, which already filters single-base substitutions, so no additional pre-filtering was required. Signature refitting was performed with the *sig_fit()* function by solving the minimization problem min(||W·H − V||) using the SBS_hg38 signature database without applying any thresholds. Sigminer performs decomposition via non-negative matrix factorization, grouping copy-number segments into feature bins (e.g., total and minor allele copy number, segment length, LOH status) and counting segments per feature category to produce a features-by-samples matrix. At the end of this process, a features × samples matrix was produced.

**MutationalPatterns**: This tool does not have any specific setting that we could experiment with. To stabilize cosine similarity calculation when comparing mutation profiles, a tiny constant of 0.0001 was added to every value in a mutational matrix.

**Sigfit**: Since the tool does not extract trinucleotide context from the VCF files, we created a custom function to generate the context matrix. We then used the built-in fit_signatures() function for mutation signature refitting. Because the MCMC algorithm is iterative, slight variation between runs is expected, so we set iter to 2000 and warm-up to 1000 after initial testing to improve precision. We retrieved signature exposures using retrieve_pars(), with hpd_prob using the highest posterior density interval—which is a Bayesian analogue to confidence intervals—set to 90%.

**Musicatk**: We used this R library for variant extraction and for building the mutational catalogue as a *musica* S4 object containing mutation counts, sample metadata and feature annotations. For signature refitting, we applied predict_exposure(), which uses the decompTumor2Sig algorithm with quadratic programming to compute non-negative signature exposures:

E_{min} || M − S × E ||^2^       subject to E ≥ 0 (1)

**SigProfilerAssignment**: SigProfilerAssignment was the only Python-based tool (python 3.9 or newer) in our workflow. Developed by the Alexandrov Lab at UC San Diego, it has been benchmarked against other methods and shown strong performance in mutational-signature assignment [9]. We performed refitting using the *Analyze.cosmic_fit()* function with the recommended parameter settings.

The following table summarizes the software tools, their key functions and settings, and the practical notes we gathered during both the pre-experimental phase and the finalization of the experimental setup.

### 2.5. Data Normalization and Tool Comparison

Normalization and comparison of matrices: The resulting matrices from each tool (Table 1) were normalized using Min–Max scaling, which maps the smallest value in each feature to 0 and the largest to 1, with all other values falling in between.

Normalized_Value = (Value − Min_Value)/(Max_Value − Min_Value)(2)

Min–max scaling was used to place the outputs of all tools on a common numerical scale. This reduces the influence of differences in absolute magnitude, total mutation burden, or tool-specific output ranges and allows the comparison to focus on the relative structure and distribution of the recovered mutational-signature profiles. Thus, scaling establishes a common basis for comparing patterns across tools using distance- and similarity-based metrics.

However, min–max scaling does not make the values equivalent to absolute mutation exposures or mutation burdens. It preserves the relative ordering within each tool’s output but removes information about between-tool magnitude differences. Therefore, the results should be interpreted as comparisons of normalized signature profiles and structural concordance, rather than direct comparisons of absolute biological activity.

To preserve consistent dimensions across tools, we added zero-value columns when signatures were missing so that all matrices shared identical column names and counts. We then compared the final matrices using the following methods: Frobenius distance, which captures squared element-wise deviations and is sensitive to large differences; Manhattan distance, which sums absolute discrepancies and is more robust to outliers; cosine similarity, which assesses directional alignment independent of magnitude; correlation, which measures shared variation patterns via Pearson’s coefficient; covariance-based distance, which compares variance–covariance structure using zero-padded covariance matrices and the Frobenius norm; and spectral distance, which evaluates global structural differences by computing Euclidean distance between singular value spectra obtained through single value decomposition.

## 3. Results

### 3.1. TRACERx 100 Experiments

We performed pairwise comparisons between the signature matrices produced by the five tools (Figure 2). For visualization purposes the distance values calculated from the normalized exposure matrices were converted to a relative similarity percentage. These percentages are visualization-specific relative similarity scores and are not absolute distance values. They are calculated using the formula:

Heatmap similarity (%) = (1 − d_ij_/d_max_) × 100 (3)

where *d_ij_* is the distance between items *i* and *j*, and *d*_max_ is the maximum observed distance. Thus, *d_ij_* = 0 gives 100% similarity, while *d_ij_* = *d*_max_ gives 0%.

### 3.2. Methods for Comparing Matrices

The selected methods for comparison of the matrices resulting from respective software tools provide complementary assessments of agreement between aligned, normalized exposure matrices rather than relying on a single definition of similarity. Frobenius and Manhattan distances quantify overall element-wise disagreement, with the former emphasizing larger deviations and the latter being less influenced by isolated extremes; cosine similarity and Pearson correlation assess agreement in profile orientation and shared variation, respectively; covariance-based distance captures differences in dependency structure among signatures; and spectral distance evaluates global matrix organization through singular-value spectra. Together, these measures assess local, distributional, and global concordance, making the comparison robust to the particular properties and limitations of any individual metric.

Distance-based measures included the Frobenius norm, which captures squared entry-wise differences, and the Manhattan norm, which sums absolute deviations. Structural similarity was evaluated using cosine similarity and Pearson’s correlation, both applied to vectorized matrices to emphasize orientation and shared variance patterns rather than magnitude.

A **covariance-based distance** quantified differences in variance–covariance structure by comparing Frobenius norm differences between aligned covariance matrices. Finally, the spectral distance measured Euclidean distances between singular value spectra, highlighting global structural differences independent of orientation.

Across **Frobenius and Manhattan distances**, musicatk and sigminer consistently clustered together, showing the smallest differences (0.805 and 58.4, respectively), while sigfit was the most divergent from all other tools (distances exceeding 35 in Frobenius and 2296 in Manhattan).

**Cosine similarity and Pearson correlation** confirmed this pattern, with musicatk and sigminer approaching perfect concordance (0.9996 correlation), while sigfit exhibited negligible similarity with any other method (similarity < 0.25). The covariance-based distance again positioned musicatk and sigminer as the closest pair (0.153), while sigfit remained an outlier (distances > 3.5).

Finally, **spectral distance** showed that musicatk and Sigminer were also the most similar in terms of global matrix structure (0.31), while sigfit deviated most strongly, reaching a maximum distance of 10.96 when compared with SigProfilerAssignment. Taken together, the six metrics support the same ranking: musicatk and Sigminer are the most concordant pair, MutationalPatterns and SigProfilerAssignment occupy an intermediate position, and sigfit is the least aligned with the rest of the toolset.

The six comparison methods showed a highly consistent pattern of agreement among the evaluated tools. Across all metrics, musicatk and Sigminer formed the closest pair, with the lowest Frobenius distance (0.0178), the lowest Manhattan distance (0.0694), the highest cosine similarity (0.9999), the highest Pearson correlation (0.9999), the lowest covariance-based distance (0.0001), and the lowest spectral distance (0.0042). These values indicate that the two tools produced nearly identical matrix structure at both the entry-wise and global levels.

MutationalPatterns was also strongly aligned with musicatk and Sigminer, although its agreement was slightly weaker. Cosine similarity remained high (0.9338 with musicatk and 0.9313 with Sigminer), as did Pearson correlation (0.9318 and 0.9293, respectively), while the corresponding distance measures remained low. This pattern places MutationalPatterns in the same broad concordant cluster, but with modest deviations in exposure magnitude or distribution.

SigProfilerAssignment showed intermediate agreement with the main cluster. It remained clearly closer to musicatk, Sigminer, and MutationalPatterns than to sigfit, but its values were consistently less concordant than those observed within the closest cluster. For example, cosine similarity ranged from 0.8296 to 0.8858 and correlation from 0.8234 to 0.8804, while Frobenius distances ranged from 0.5701 to 0.6865. This suggests that SigProfilerAssignment captures a related overall signal, but with more noticeable shifts in matrix composition.

In contrast, sigfit was the clear outlier across all six methods. It showed the largest Frobenius distances (1.7784–1.9086), the largest Manhattan distances (5.1621–5.5588), the weakest cosine similarities (0.0310–0.1573), and the weakest correlations (−0.0176 to 0.1057). The covariance-based and spectral comparisons led to the same conclusion. The near-zero and slightly negative correlation with some tools indicates that sigfit did not preserve the same variation pattern observed in the other methods.

Taken together, the rankings were stable across all six comparison approaches (Figure 3): musicatk and Sigminer showed the strongest concordance, MutationalPatterns remained closely aligned with this pair, SigProfilerAssignment occupied an intermediate position, and sigfit was consistently the most divergent tool. This agreement across mathematically distinct metrics indicates that the observed pattern is robust and does not depend on a single similarity measure.

Because the ranking was reproduced across metrics that emphasize different mathematical properties, the observed agreement cannot be explained by a single numerical artifact. Instead, it points to a robust methodological split between deterministic refitting frameworks and the posterior-based solution returned by sigfit.

### 3.3. Genome in a Bottle Experiments

To ensure our findings were not driven solely by the large, multi-sample TRACERx exome dataset, we repeated the entire comparison using the single-sample whole-genome dataset GIAB. Unlike TRACERx 100, GIAB’s simpler structure allowed us to test whether the cross-tool ranking remains consistent when applied to a minimal dataset.

GIAB comparison (Figure 4) reproduced the same qualitative ordering observed in TRACERx: musicatk and Sigminer remained the closest pair, MutationalPatterns and SigProfilerAssignment showed intermediate agreement, and sigfit remained the most distinct solution. This consistency strengthens the conclusion that the ranking reflects algorithmic behavior rather than only the size or heterogeneity of the input cohort.

At the same time, the absolute magnitudes of the GIAB distances were smaller overall. That result is expected for a single-sample dataset, where there is less between-sample variability and therefore fewer opportunities for disagreement to accumulate across the matrix. The preservation of ranking together with the reduction in scale indicates that dataset complexity amplifies disagreement but does not fundamentally change which tools behave similarly.

Comparison of the two datasets showed that the ranking of tool similarity was consistent across both datasets. This indicated that the overall tool performance was not biased by the dataset. However, we also observed that the magnitude of distances differs. The distances in GIAB were smaller overall, likely because GIAB is a single-sample dataset (less variability) whereas TRACERx is multi-sample (427 WES samples).

Across the normalized signature matrices, GIAB showed slightly lower per-sample cross-tool distances than TRACERx (Table 2). The mean Frobenius and Manhattan distances were 11.3% and 18.1% lower in GIAB, respectively, while cosine similarity and Pearson correlation were 5.0% and 6.7% higher. These results suggest that the greater cross-tool variation observed in TRACERx is partly associated with the complexity and heterogeneity of its multi-sample structure. However, because GIAB contains only one sample, this comparison cannot separate the effect of sample number from other dataset-specific factors and should therefore be interpreted descriptively rather than as a formal statistical comparison.

Covariance-based and spectral distances were slightly higher for GIAB than for TRACERx. However, the relative increases were driven by the small TRACERx baseline, and these metrics should be interpreted alongside their absolute differences and the other similarity measures. For GIAB, values represent averages across the 10 tool pairs for its single sample.

### 3.4. Biological Interpretation and Cross-Tool Comparison of Mutational Signatures

The extracted signatures showed variability between tools. In the TRACERx data, SBS5 was among the main signatures identified by four of the five tools: musicatk, MutationalPatterns, SigMiner, and SigProfiler. SigFit instead ranked SBS25 first and did not include SBS5 among its three highest-mean signatures. In the GIAB data, SBS5 was also identified among the main signatures by four tools, whereas SigFit again ranked SBS25 first.

SBS5 is a broadly distributed, clock-like signature detected across many cancer types (Figure 5). Its activity is generally associated with endogenous, age-related mutagenesis, although it may also reflect additional processes and can be difficult to distinguish from SBS16. Increased SBS5 burden has been reported in lifelong smokers compared with never-smokers, with a 1.3–5.1-fold difference, and in bladder tumours with ERCC2 mutations [13,14]. Therefore, SBS5 should not be interpreted as a specific smoking signature without additional evidence.

SBS1 was also commonly recovered. It is a clock-like signature associated primarily with age and cell division. It results from spontaneous or enzymatic deamination of 5-methylcytosine, producing C > T substitutions. If the resulting G:T mismatches are not repaired before DNA replication, the mutation becomes fixed. The rate at which SBS1 mutations accumulate varies between cancer types and normal cell types and is related to tissue-specific stem-cell division rates [13]. SBS1 was among the main signatures identified by four TRACERx tools and by several tools in GIAB, although its ranking and mean value varied.

The TRACERx results did not show SBS4, SBS29, or SBS92 among the three highest-mean signatures for any tool [15]. SBS4 is the signature most directly associated with tobacco smoke exposure [16]. It is characterized predominantly by C > A transversions, also described as G > T substitutions because of damaged guanine, and is linked to carcinogens such as benzoapyrene in tobacco smoke. SBS4 is common in lung cancer and can also occur in head and neck cancer. SBS7, which is associated with ultraviolet-induced DNA damage, was likewise not among the dominant signatures in the retained results. However, the absence of these signatures from the top-three ranking does not demonstrate that their exposures were zero, because the analysis used min–max-scaled matrices rather than absolute exposure estimates.

The differences between tools were large enough to change the ranking of the dominant signatures, particularly for SigFit, which prioritized SBS25 and assigned substantially higher relative values to SBS37 and SBS39 than the other tools. Nevertheless, the retained results do not allow us to determine whether these differences would change biological conclusions such as classification of homologous recombination deficiency (HRD) or mismatch-repair deficiency (MMR-D). No HRD- or MMR-D-specific classifier, threshold, absolute exposure table, or uncertainty estimate was available. Consequently, the present analysis demonstrates methodological differences in signature representation but does not establish clinically meaningful disagreement in HRD or MMR-D status.

Several limitations should be noted. The study compares normalized matrices rather than biological exposure tables. Min–max scaling places features on a common <0, 1> range but does not normalize mutation burden or signature exposure. Heatmap percentages are range-normalized displays and are therefore not numerically equivalent to the absolute distances reported in the text. SigFit should not be considered inferior solely because it diverges from the other tools, since its posterior-based inference represents uncertainty differently from point-estimate workflows.

The present analysis was designed to evaluate concordance between mutational-signature profiles generated by different tools. Accordingly, the reported rankings and values should be interpreted as relative comparisons of SBS profiles, rather than as direct estimates of absolute mutation burden or clinical status. The analysis focused on single-base substitutions; indel signatures were outside its scope, and multiallelic SBS events were handled separately. The observed differences in signature ranking identify candidates for further validation using repeated analyses, uncertainty estimates, and clinically annotated HRD and MMR-deficiency classifications. These analyses would help determine whether methodological differences in signature exposure estimates translate into different biological or clinical conclusions.

## 4. Discussion

In this study, we show that even when applied to identical inputs with identical preprocessing, the five NMF-based signature extraction tools produce notably different outputs. Musicatk and sigminer consistently aligned across all similarity and distance metrics, indicating near-equivalent results and suggesting that their NMF implementations yield highly reproducible factorization patterns. Mutpatterns and SigProfiler showed moderate agreement, whereas sigfit diverged systematically from all other methods, implying substantial differences in its optimization or regularization strategy. Because preprocessing and mutational processes were controlled, these discrepancies must stem from algorithmic design and parameterization. Our goal was to assess whether current tools can deliver concordant and biologically plausible results from the same input; the findings indicate that some implementations—particularly musicatk and sigminer—are more reliable for mutational-signature analysis, while others may require additional scrutiny before use in comparative or integrative studies.

The aim of the study was to compare the outputs produced by the evaluated tools and to determine how closely their results agreed under the same input conditions, rather than to quantify the statistical uncertainty or run-to-run stability of each method. Accordingly, the analysis was based on representative point estimates and did not include repeated runs, bootstrapping, or confidence and credible intervals. The observed divergence of sigfit should therefore not be interpreted as evidence of inferior performance. Sigfit uses a Bayesian MCMC framework and represents exposures through posterior distributions, whereas the other tools primarily return deterministic point estimates. Its greater distance from the other outputs may thus partly reflect a different statistical approach and treatment of uncertainty. In the absence of a validated ground truth, the present results describe cross-tool concordance but cannot establish which tool is more accurate. A systematic assessment of stability and uncertainty remains an important direction for future experiments. Taken together, our results show that cross-tool concordance offers useful insight into the relative robustness of signature extraction methods, but concordance alone cannot guarantee biological validity. Establishing whether a tool can truly recover underlying mutational processes requires further benchmarking with simulated datasets, stability testing through cross-validation, and integration of biological prior knowledge. Ensemble strategies that combine outputs from multiple tools may also help mitigate method-specific biases and strengthen the reliability of mutational-signature discovery. Developing such best practices will be essential for ensuring that signature extraction yields results that are not only consistent but also biologically meaningful.

## 5. Conclusions

The central objective of this experiment was to compare the outputs generated by different mutational-signature refitting tools under the same experimental conditions. Filtering and preprocessing were applied to remove technical artefacts and to make the input data as consistent and reliable as possible. Although this procedure cannot guarantee a perfectly clean or biologically complete dataset, its limitations do not undermine the main comparative principle: all tools were applied to the same input data. Therefore, any systematic differences in the resulting signature exposures or rankings cannot reasonably be attributed solely to differences in the underlying mutation set.

If the tools implemented equivalent procedures, the use of identical input data would be expected to produce highly similar results, even if the input itself retained some imperfections. Consequently, the observed discrepancies provide evidence of differences in software implementation, model formulation, optimization strategy, or internal data handling. The experiment should therefore be interpreted as a controlled assessment of tool-dependent variability rather than as an attempt to establish one output as an absolute biological truth. Concordance can indicate relative robustness, but it cannot guarantee correctness; systematic benchmarking and cross-tool validation remain necessary for reliable biological interpretation.

At present, there is no universally accepted “gold standard” tool for mutational signature extraction that could serve as a definitive reference for comparison. Each available method is based on its own implementation of non-negative matrix factorization or related approaches, with differences in optimization routines, regularization, and parameterization. Consequently, concordance between tools can indicate relative robustness, but it does not guarantee the results are correct, since none of the methods can be assumed to yield the true underlying mutational signatures. This lack of a reference standard means that no single tool can be relied upon in isolation, underscoring the need for systematic benchmarking, cross-tool validation, and ensemble strategies to approach reliable biological interpretation.

## Figures and Tables

**Figure 1:**
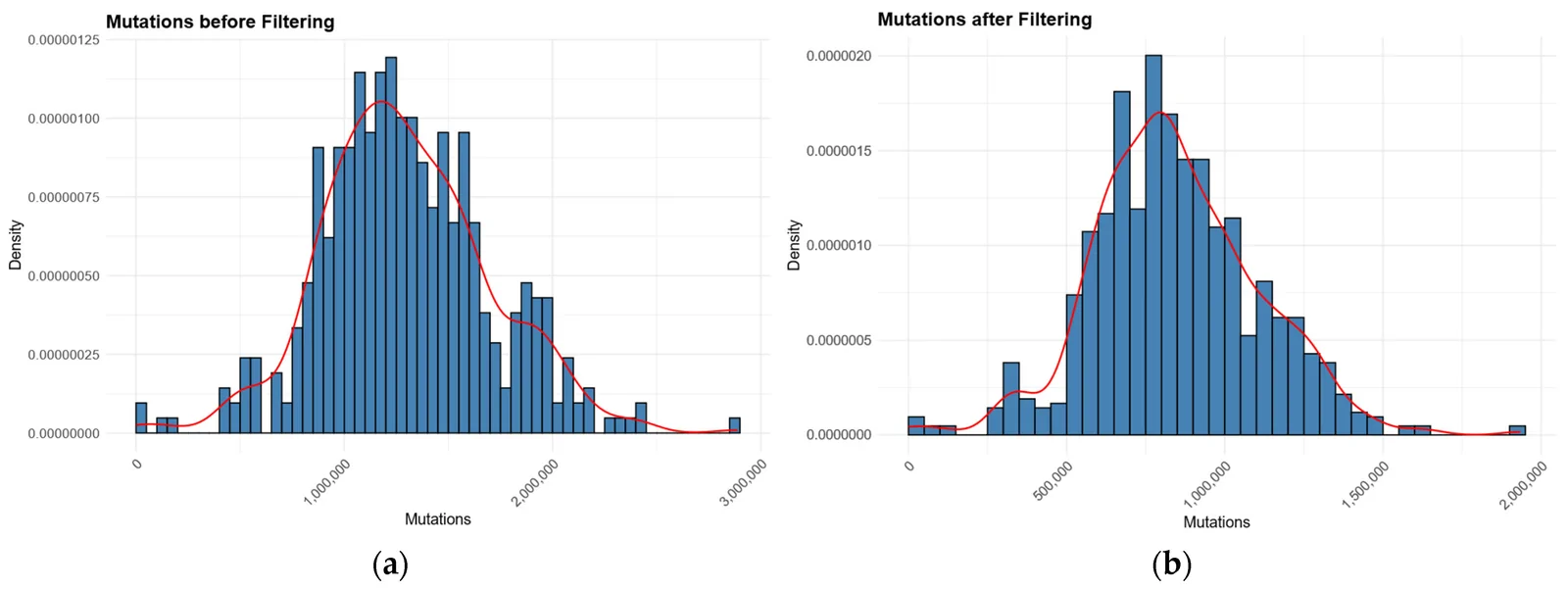
(**a**) Histogram of mutations in TRACERx dataset before filtering with median = 1,259,914, min. = 845 and max. = 2,888,355; (**b**) Histogram of mutations after filtering with median = 815,684, min. = 312 and max. = 1,933,802.

**Figure 2:**
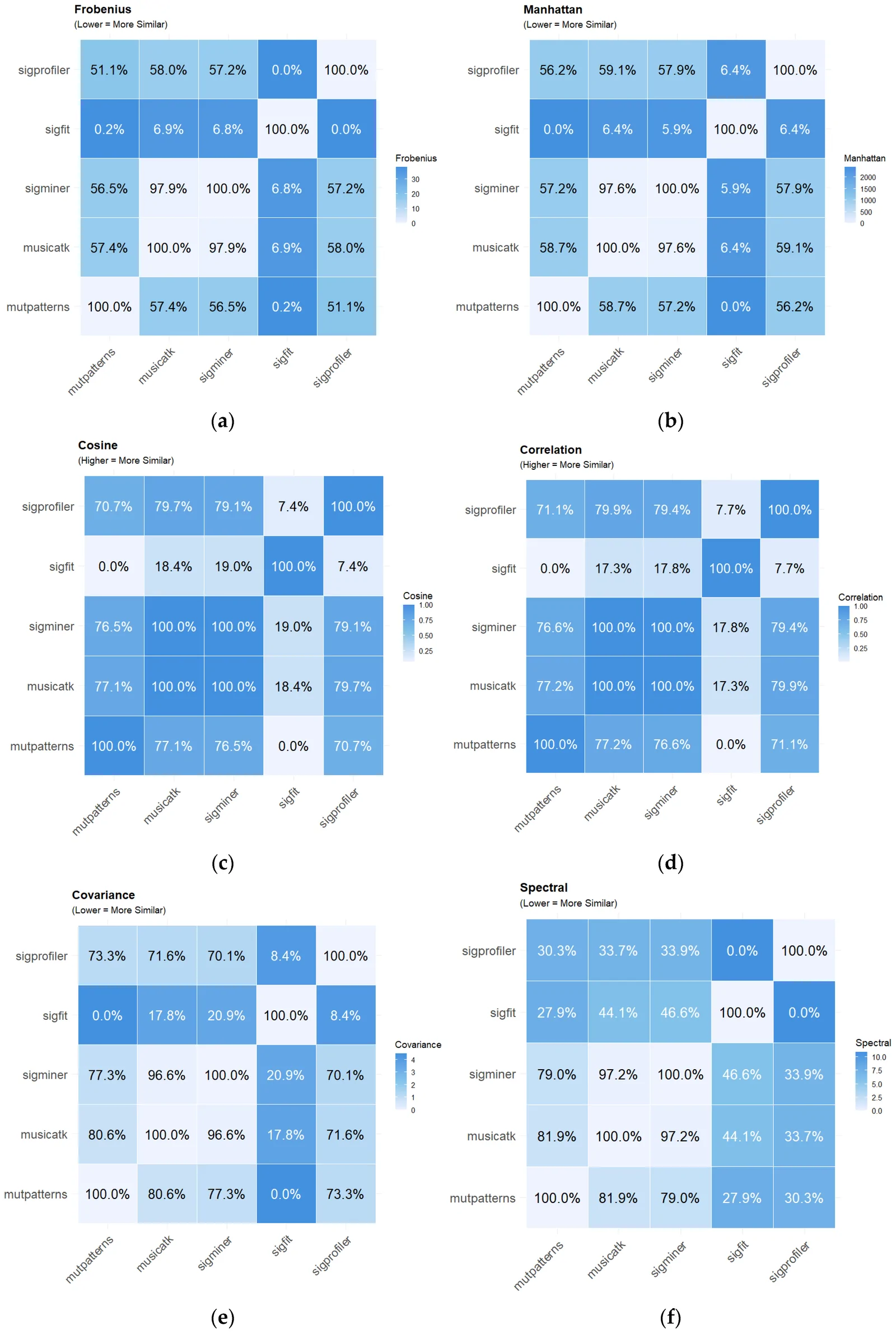
Pairwise comparison heatmaps for the five refitting tools applied to the TRACERx dataset. Panels (**a**–**f**) show Frobenius distance, Manhattan distance, cosine similarity, Pearson correlation, covariance-based distance, and spectral distance, respectively. Rows and columns represent tools; diagonal cells are self-comparisons and off-diagonal cells are pairwise comparisons. For distance metrics, lower values indicate greater agreement, whereas for cosine similarity and Pearson correlation, higher values indicate greater agreement. The consistently close musicatk-Sigminer cells and the comparatively distant sigfit cells identify the principal concordant cluster and outlier across the six metrics.

**Figure 3:**
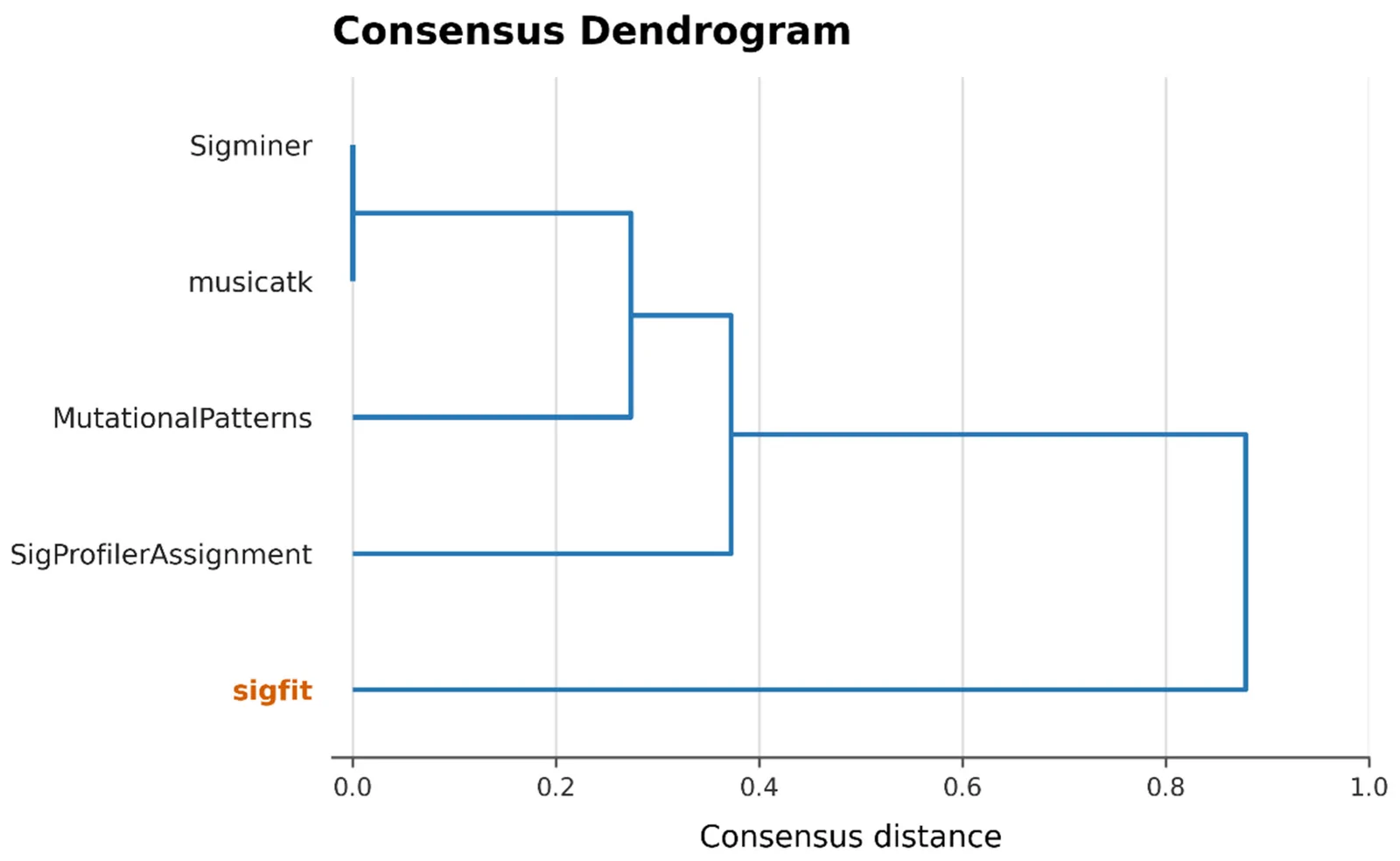
Consensus dendrogram of the five mutational-signature refitting tools. Pairwise comparisons from the six metrics were converted to a common distance scale, where lower values indicate greater similarity. Cosine similarity and Pearson correlation were transformed using *distance = 1 − similarity*, after which each distance matrix was normalized to the <0, 1> range and the six matrices were averaged with equal weights to obtain a consensus distance matrix. Average-linkage hierarchical clustering was then applied to this matrix. Tools that merge at lower consensus distances produce more similar results across the evaluated metrics. Musicatk and Sigminer formed the closest pair, whereas sigfit joined the remaining tools at the highest consensus distance and was therefore the most distinct.

**Figure 4:**
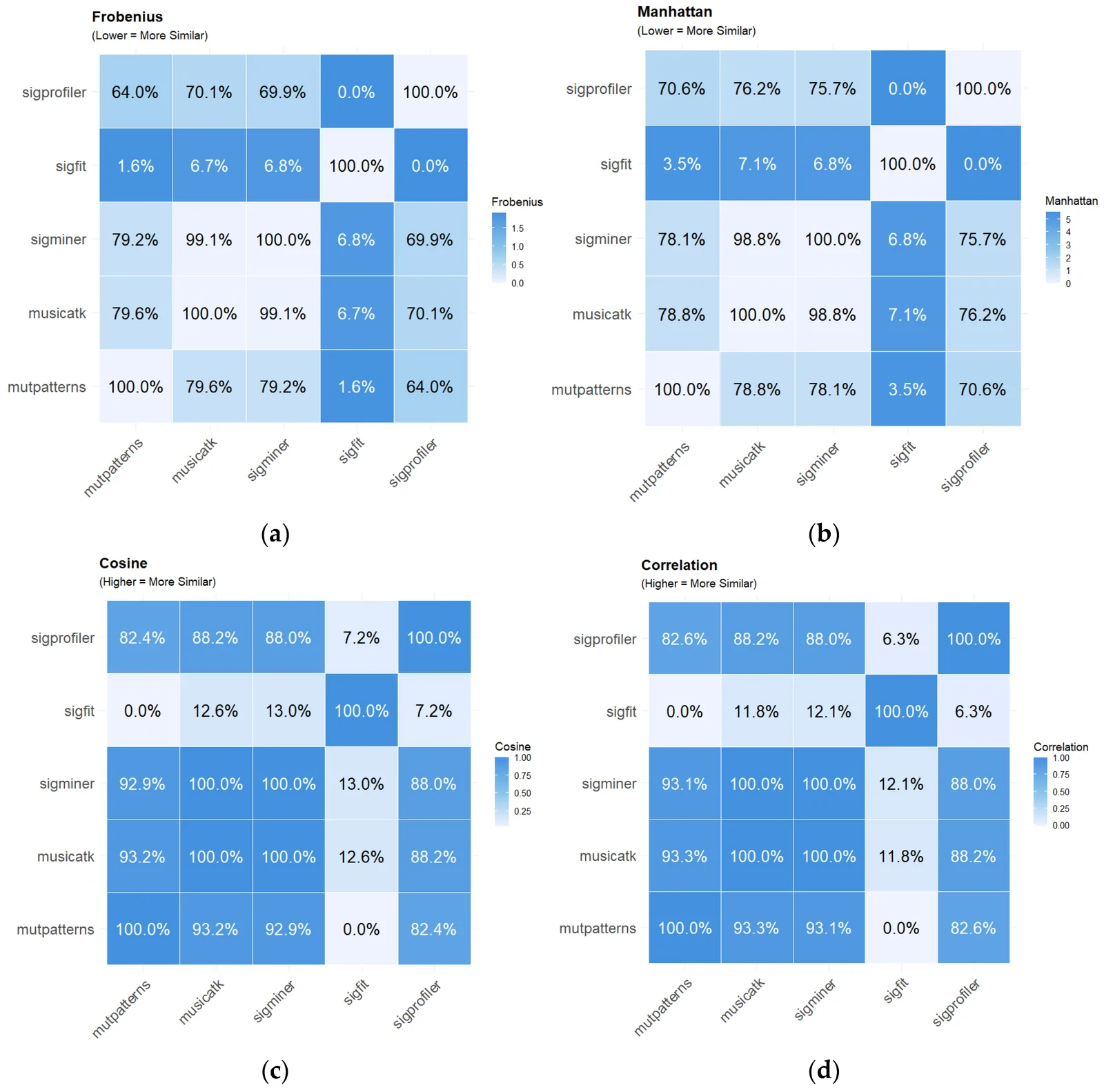

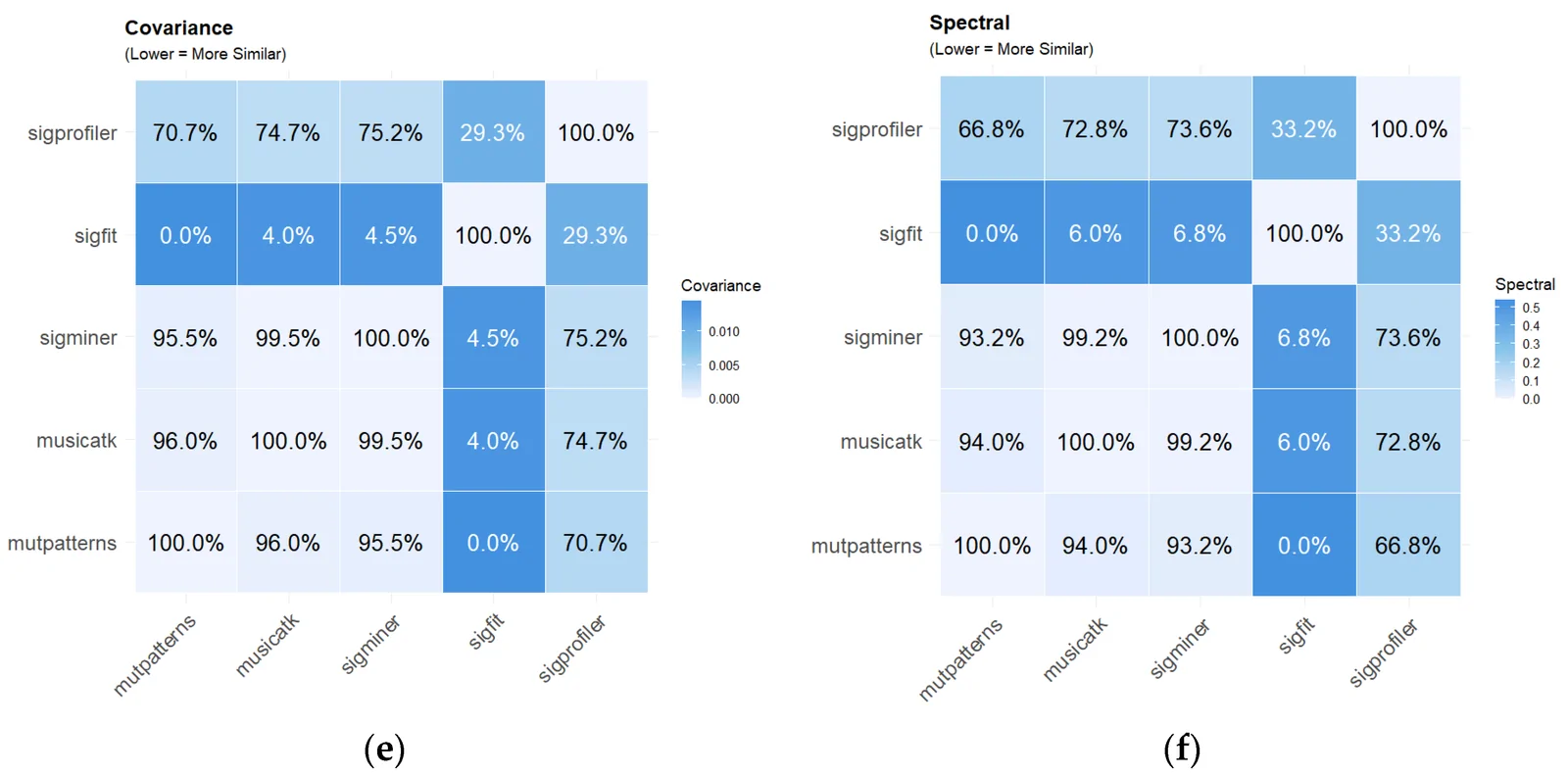
Pairwise comparison heatmaps for the five refitting tools applied to the Genome in a Bottle dataset. Panels (**a**–**f**) show Frobenius distance, Manhattan distance, cosine similarity, Pearson correlation, covariance-based distance, and spectral distance, respectively. Rows and columns represent tools, and each off-diagonal cell gives the corresponding pairwise comparison. Lower values indicate greater agreement for the four distance metrics, while higher values indicate greater agreement for cosine similarity and Pearson correlation. The repeated musicatk-Sigminer proximity and the separation of sigfit from the other tools show that the qualitative ranking is retained in this single-sample whole-genome test, despite its smaller absolute distances.

**Figure 5:**
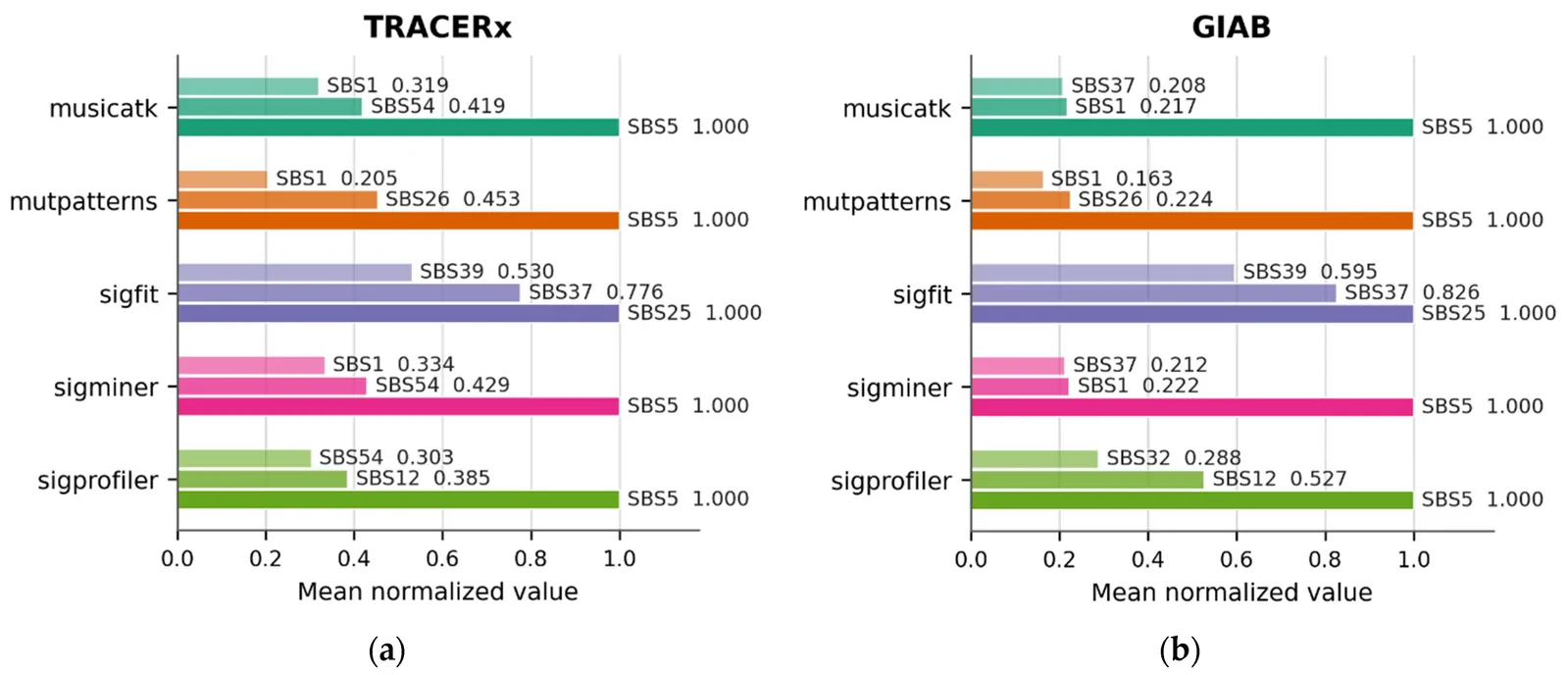
(**a**) Mean values of the three most active mutational signatures identified by each tool in the GIAB dataset; (**b**) mean values of the three most active mutational signatures identified by each tool in the TRACERx dataset. Values were normalized to the interval <0, 1>. Bars are labelled with the corresponding COSMIC SBS signature and normalized value.

**Table 1:** Comparison of the tools used for refitting calculations.

Tool	Refitting Algorithm	Main Function (s)	Notes on Precision and Effectiveness
Sigminer	Quadratic Programming/NNLS	sig_tally, sig_fit	Deterministic; provides sample-wise refitting; precision depends on input data and signature selection.
MutationalPatterns (R)	NNLS	fit_to_signatures	Reliable; strict mode improves stability; bootstrapping allows assessment of confidence in exposures.
Sigfit (R)	Bayesian MCMC	fit_signatures	Probabilistic; provides posterior distributions (uncertainty estimates); slower but gives richer information about exposure confidence.
MusicaTk (R)	NNLS/EM	predict_exposure	Flexible; supports multiple algorithms; precision depends on mutation counts and selected method.
SigProfilerAssignment (Python)	Forward Stagewise + NNLS	cosmic_fit	High precision; optimized for assigning known COSMIC signatures; avoids spurious low contributions.

**Table 2:** Quantitative comparison of tool performance on GIAB and TRACERx.

Metric	TRACERx: Mean Across Samples and Tool Pairs	GIAB: Mean Across Tool Pairs for One Sample	GIAB Change Relative to TRACERx
Frobenius distance	1.125601	0.997956	11.3% decrease
Manhattan distance	3.425409	2.803890	18.1% decrease
Cosine similarity	0.562467	0.590657	5.0% increase
Pearson correlation	0.532182	0.567727	6.7% increase
Covariance-based distance	0.004804	0.006634	38.1% increase
Spectral distance	0.174244	0.245966	41.2% increase

## Data Availability

The TRACERx dataset analyzed in this study is available through the European Genome-phenome Archive (EGA), subject to the repository’s access requirements. No new datasets were generated during this study.
